# In Vitro Effects of Titanium Dioxide Nanoparticles (TiO_2_NPs) on Cadmium Chloride (CdCl_2_) Genotoxicity in Human Sperm Cells

**DOI:** 10.3390/nano10061118

**Published:** 2020-06-05

**Authors:** Marianna Santonastaso, Filomena Mottola, Concetta Iovine, Fulvio Cesaroni, Nicola Colacurci, Lucia Rocco

**Affiliations:** 1 Department of Woman, Child and General and Special Surgery, University of Campania “Luigi Vanvitelli”, 80138 Napoli, Italy; marianna.santonastaso@unicampania.it (M.S.); Nicola.colacurci@unicampania.it (N.C.); 2 Department of Environmental, Biological and Pharmaceutical Sciences and Technologies, University of Campania “Luigi Vanvitelli”, 81100 Caserta, Italy; filomena.mottola@unicampania.it (F.M.); concetta.iovine@unicampania.it (C.I.); 3 PMA Center of Cassinate, 03043 Cassino, Italy; fulviocesaroni@hotmail.it

**Keywords:** sperm DNA damage, titanium dioxide nanoparticles, cadmium, oxidative stress, male infertility

## Abstract

The environmental release of titanium dioxide nanoparticles (TiO_2_NPs) associated with their intensive use has been reported to have a genotoxic effect on male fertility. TiO_2_NP is able to bind and transport environmental pollutants, such as cadmium (Cd), modifying their availability and/or toxicity. The aim of this work is to assess the in vitro effect of TiO_2_NPs and cadmium interaction in human sperm cells. Semen parameters, apoptotic cells, sperm DNA fragmentation, genomic stability and oxidative stress were investigated after sperm incubation in cadmium alone and in combination with TiO_2_NPs at different times (15, 30, 45 and 90 min). Our results showed that cadmium reduced sperm DNA integrity, and increased sperm DNA fragmentation and oxidative stress. The genotoxicity induced by TiO_2_NPs-cadmium co-exposure was lower compared to single cadmium exposure, suggesting an interaction of the substances to modulate their reactivity. The Quantitative Structure-Activity Relationship (QSAR) computational method showed that the interaction between TiO_2_NPs and cadmium leads to the formation of a sandwich-like structure, with cadmium in the middle, which results in the inhibition of its genotoxicity by TiO_2_NPs in human sperm cells.

## 1. Introduction

Titanium dioxide is a colorless, crystalline and poorly soluble powder. The small dimensions of the crystals are responsible for their particular physical–chemical characteristics and enhanced reactivity, unlike other solid materials and larger particles with the same chemical composition [1,2]. Titanium dioxide used in the form of nanoparticles (TiO_2_NPs) showed different properties such as robust oxidation, biocompatibility and photocatalysis. Therefore, TiO_2_NPs are used in a wide range of applications, including pharmaceuticals, cosmetics, paints, medicine and engineering. However, there are some concerns about the possible biological effects associated with their use [3]. In fact, the increased use of TiO_2_NPs in industry and in daily applications (domestic, cosmetic, food) has attracted growing interest because, to date, we cannot yet accurately predict and control the impact on health due to their release into the environment. It is known that TiO_2_NPs induce in vivo and in vitro genotoxicity and cytotoxicity on several experimental models, by altering the genome stability, increasing the apoptosis and decreasing the cell viability in different vertebrates [4,5,6,7].

TiO_2_NPs negatively influence male fertility, as they lead to a reduced sperm quality and daily sperm production, reduced weight of the testes and histopathological testicular changes. A review on the reproductive and developmental toxicity of nanomaterials indicates that the studies are generally performed in adult or pre-pubertal/pubertal rats or mice [8]. Recently, it has been shown that TiO_2_NPs have adverse effects on human reproduction by inducing DNA sperm damage. Human ejaculated spermatozoa treated with TiO_2_NPs showed a loss of DNA integrity, probably due to the production of intracellular reactive oxygen species (ROS) [9].

In addition to toxicity and genotoxicity caused by their inherent and unique properties [10,11], TiO_2_NPs were also demonstrated to interact with pollutants, either organic or heavy metals, modulating bioaccumulation and toxic responses in co-exposed organisms, and modifying their fate, behavior, bioavailability and toxicity for the ecosystem and human health [12,13]. In fact, TiO_2_NPs were able to phagocytize and carry other pollutants and/or drugs, hence skipping the natural cellular defenses, through the mechanism known as the “Trojan Horse effect” [14,15,16]. However, the interaction between TiO_2_NPs and co-existing contaminants in the environment remains unclear, with conflicting results. On human amniocytes in vitro, TiO_2_NPs increased the genotoxicity of lincomycin through a loss of DNA integrity, apoptosis and DNA damage [17]. In zebrafish larvae, *Daphnia magna* and carp, TiO_2_NPs enhanced lead (Pb), copper (Cu), arsenic (As) (III), zinc (Zn) and cadmium (Cd) bioaccumulation and toxicity [18,19,20,21,22], while, in algae (*Chlamydomonas reinhardtii* and *Microcystis aeruginosa*) and amphipods (*Gammarus fossarum*), TiO_2_NPs reduced the bioavailability and toxicity of Cd and Cu [23,24,25]. TiO_2_NPs enhanced the Cd and nickel (Ni) reproductive and developmental toxicity in *Caenorhabditis elegans* in a dose-dependent manner [26]. Among heavy metal, Cd is the most widespread in industrial applications, ranked as the seventh most toxic heavy metal, with a specific toxicological profile (ATSDR 2012) that describes its adverse effects for living organisms and human health [27]. Due to its application in fertilizers, battery, pigments and plastics [28], Cd may enter the natural environment and impact human health and the environment [29]. Cd and its compounds were classified as type I human carcinogens in 1993 by the International Agency for Research on Cancer, IARC [30]. Cd is reported as toxic for organs, such as the kidney, liver and stomach, causing respiratory and bone disease, as well as neurological disorders [31,32,33]. Finally, Cd exerts negative effects on human reproduction. In fact, Cd concentration in the human seminal plasma is closely related to working conditions, food and cigarette smoke, with a reduced fertility being observed in highly exposed patients [34]. In vitro studies have shown that Cd affects sperm motility and the sperm’s ability to reach and penetrate into the oocyte [35,36,37,38]. Furthermore, rats exposed to Cd showed a reduced testicular volume sperm concentration and testosterone concentration in the Leydig cells, as well as an increased follicle-stimulating hormone (FSH) concentration in the serum [39].

Cd-induced damage depends on the dose, duration of exposure, type of contact, as well as the interaction with other materials and/or nanomaterials. The effects of TiO_2_NPs and Cd^2+^ co-exposure have been investigated in plants and aquatic species [6,40,41,42,43,44,45,46]. A recent study showed that a non-cytotoxic concentration of TiO_2_NPs enhanced the toxicological potential of Cd^2+^ in human liver (HepG2) and human breast cancer (MCF-7) cells [47]. However, the overall results are conflicting, underlining that the influence of TiO_2_NPs on Cd^2+^ accumulation and toxicity varies according to the species, tissues, culture media and physical–chemical behavior of particles in exposure media. Moreover, their influence on reproductive health is still scarcely investigated.

This study aimed to evaluate the genotoxicity of Cd and to investigate the combined effects of TiO_2_NPs and cadmium chloride (CdCl_2_) on human ejaculated sperm cells in vitro. In this study, we attempted to determine whether TiO_2_NPs could modify the possible Cd genotoxic responses. To achieve our goal, we investigated cytotoxicity, genotoxicity, oxidative stress and apoptosis in human sperm cells after TiO_2_NPs and CdCl_2_ co-exposure. To our knowledge, this is the first study to evaluate the responses of human sperm on CdCl_2_ and TiO_2_NPs co-exposure. It provided new insights into the TiO_2_NPs’ interaction with heavy metal and clarified the potential reproductive health risk of manufactured nanoparticles as carriers of contaminants.

## 2. Material and Methods

### 2.1. Chemicals

Nano-powder of TiO_2_NPs (Aeroxide) was supplied by Evonik Degussa (Essen, Germany; Lot. 614061098). Aeroxide is a mixture of 75% rutile and 25% anatase forms with an average primary particle size of 21 nm and declared purity of 99.9%. CdCl_2_ (CAS number 10108-64-2, 99.999% purity) was acquired from Sigma-Aldrich (St. Louis, Missouri, USA). CdCl_2_ was dissolved in water to a stock concentration of 100 mM. Benzene (CAS number 71-43-2, 99.8 purity) was provided from Sigma-Aldrich (St. Louis, MO, USA).

### 2.2. Preparation and Characterization of TiO_2_NPs

**Figure 1:**
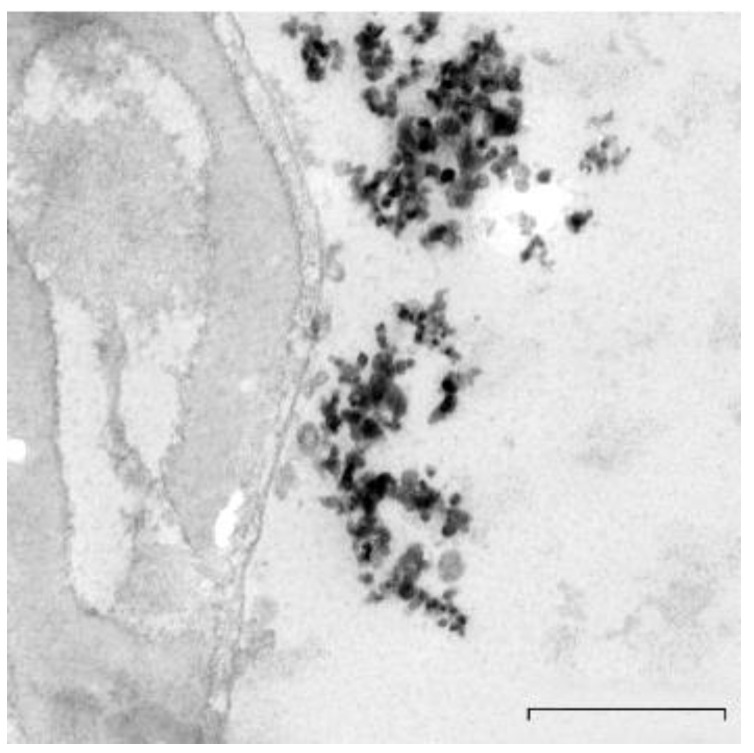
TEM micrograph showing the aggregation pattern of TiO_2_ nanoparticles (bar 0.5 µm).

TiO_2_NPs stock solutions (10.0 mg/L) were prepared by dispersing the NPs in Eagle’s Minimum Essential Medium (MEM) with sonication (40 kHz frequency, Dr. Hielscher UP 200S, Teltow, Germany) and were dosed according to Santonastaso and collaborators [9]. Briefly, we acquired UV-Vis spectra of TiO_2_NPs in the range of 200–600 nm using a Shimadzu UV-1700 double-beam spectrophotometer. UV spectra of 1 μg/L TiO_2_NPs-enriched culture medium at 15, 45 and 90 min showed a secondary peak at longer wavelengths probably due to the formation of agglomerates (Table 1). The primary particle diameter and shape were determined by Zeiss-LIBRA120 (Carl Zeiss Oberkochen, Germany) Transmission Electron Microscope (TEM). TiO_2_NPs’ TEM images showed a size distribution ranging approximately from 20 to 60 nm, with a partly irregular and semispherical shape, and agglomeration occurred with a diameter in the range of 400 ± 52 nm (Figure 1).

### 2.3. Sample Collection, Evaluation and Exposure Procedure

Semen samples were obtained by masturbation from 125 men between 25 and 39 years old, underwent routine semen analysis and were examined in our Reproduction Biology Laboratory (University of Campania L. Vanvitelli). Patients were informed about the purpose of the study and they signed written informed consent. Subjects on any medication or antioxidant supplementation were not included. All ejaculates presenting normal semen parameters with a seminal white blood cell count <0.5 × 10^6^ /mL, which was less than the pathologic concentration, were used in the study (Table 2) [48]. After liquefaction at room temperature for 30 min, the semen volume, pH, sperm concentration, motility, morphology and viability were determined according to the World Health Organization (WHO) guidelines (2010) [48]. The percentage of morphologically abnormal spermatozoa was evaluated by Test-simplets^®^ pre-stained slides (Origio, Cooper Surgical, Inc., Trumbull, CT, USA). Sperm vitality was assessed by the eosin–nigrosine staining. The ejaculates were purified by discontinuous density gradient centrifugation. The sperm preparation was done using a 45%–90% double density gradient (SPERM GRAD™; Vitrolife, Göteborg, Sweden) in order to obtain a sufficient number of selected spermatozoa to perform the experiments.

Each purified sample was divided into four aliquots (1 × 10^6^ sperm/mL). One aliquot was exposed to 10 µM of CdCl_2_, another aliquot was co-exposed to 1 µg/L of TiO_2_NPs and 10 µM of CdCl_2_; one aliquot was treated with 0.4 µL/mL benzene as positive control [49], while an untreated aliquot was used as negative control each time. TiO_2_NPs and CdCl_2_ concentrations were selected based on previous in vitro studies [9,36]. The exposition was evaluated after 15, 30, 45 and 90 min (min). Incubation was performed in Eagle’s Minimum Essential Medium (MEM) at 36.5 °C. After exposure, the samples were centrifuged for 5 min at 1500 rpm, the supernatant was removed, the pellet was re-suspended in 500 µL bicarbonate buffer and the semen parameters were evaluated.

### 2.4. Comet Assay

**Figure 2:**
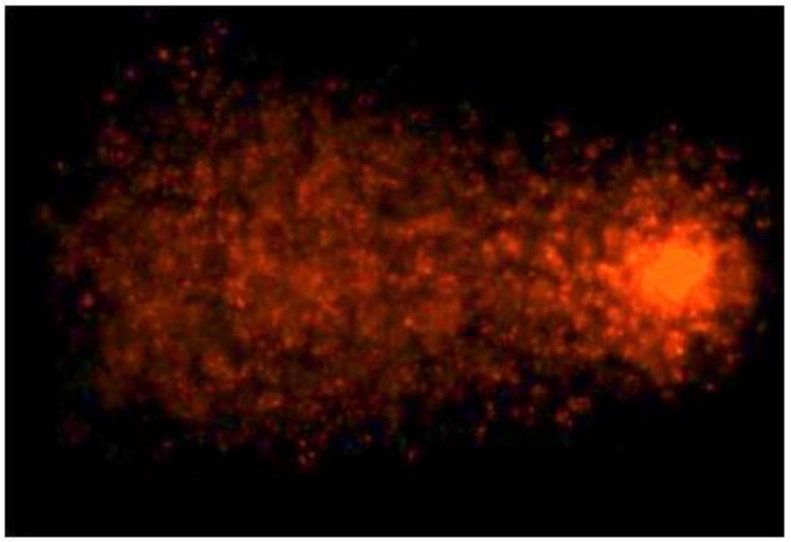
Comet tail DNA in human sperm cell analyzed using “OpenComet” software.

The Comet assay and the relative statistical analyses were performed according to Santonastaso and collaborators [9]. Briefly, sperm cells were mixed with low melting point (LMP, 0.7%) agarose and were included into normal melting point (NMP, 21%) agarose layers on slides for 30 min. Then, another LMP layer was added. The slides were treated with lysis buffer (NaCl 2.5 M, Na_2_EDTA 0.1 M, Tris-Base 0.4 M, Triton X-100 1%, dimethyl sulfoxide (DMSO) 10%, pH 10) and were enzymatically digested with proteinase K (0.5 µg/L). After washing with a neutralizing solution, the slides were incubated in alkaline buffer (NaOH 10N, EDTA 200 mM, pH 12.1) for 10 min and then exposed to electrophoresis (25 V, 300 mA, 20 min). After fixing in cold methanol and staining with ethidium bromide, the slides were observed by using a fluorescence microscope with 60X magnification (Nikon Eclipse E-600). The images were acquired by means of the “OpenComet” software [50]. The Comet assay was performed in triplicate. The parameter that was considered was the percentage of damaged DNA in the comet tail (% Tail DNA) (Figure 2).

### 2.5. TUNEL Test

The TUNEL test was performed according to Santonastaso and collaborators [9] using the In-Situ Cell Death Detection Kit (Roche Diagnostics, Basel, Switzerland). The sperm samples were put on glass slides, fixed in 4% paraformaldehyde for 1 h and air-dried. After 2 min incubation in a permeabilizing solution, the slides were washed in bicarbonate buffer and air-dried. Then, 5 µL of terminal deoxy nucleotidyl transferase enzyme solution and 45 µL of label solution were placed on each slide. After 1 h incubation in a humid chamber at 37 °C, the slides were stained in 4′,6-diamidino-2-phenylindole (DAPI) solution for 5 min, and 100 µL of 1,4 diazobicyclo (2,2,2) octane (DABCO) solution (20×) was added to each slide. The TUNEL test was performed in triplicate. The slides were analyzed by using a fluorescence microscope (Nikon Eclipse E-600) equipped with BP 330–380 nm and LP 420 nm filters. The percentage of sperm with fragmented DNA was referred to as the percentage of TUNEL-positive sperm.

### 2.6. Genomic DNA Extraction, RAPD-PCR Technique and Genomic Template Stability

Sperm cells’ DNA was extracted and purified from 200 µL/sample, using the High pure PCR template preparation Kit (ROCHE Diagnostics^®^). The RAPD method is a PCR-based technique that amplifies random DNA fragments with the use of single short primers under low annealing conditions. The PCR amplifications were performed in 25 μL of reaction mixture containing 40 ng of DNA, 5 pmoL/µL of primer D11 (5′-d[GTCCCGACGA]-3′) and Taq DNA recombinant polymerase (Roche Diagnostics, Basel, Switzerland), including nucleotides (dNTPs 0.4 mM), magnesium chloride and DNA polymerase. D11 primer was selected to yield amplification products with a reasonable number of bands [9]. The PCR program consisted of an initial denaturation at 94 °C for 2 min, then 45 cycles, each of them including DNA denaturation at 95 °C for 1 min, annealing at 36 °C for 1 min and extension at 72 °C for 1 min. The reaction products were analyzed by an electrophoretic run on 2% agarose gel and gel staining with 10× ethidium bromide. RAPD-PCR patterns were acquired by ChemiDoc Gel Imaging System (Bio-Rad, Hercules, CA, USA). The change in the number of the bands and the variation in their intensity are associated with alterations of genetic material [51]. The polymorphic pattern generated by the RAPD-PCR profiles allowed the calculation of the Genomic Template Stability (GTS, %) as follows: GTS% = (1 − a/n) × 100, where a is the average number of polymorphic bands detected in each exposed sample, and n is the number of total bands in the non-treated cells. The template genomic stability of the control was set to 100% [52].

### 2.7. DCF Assay

**Figure 3:**
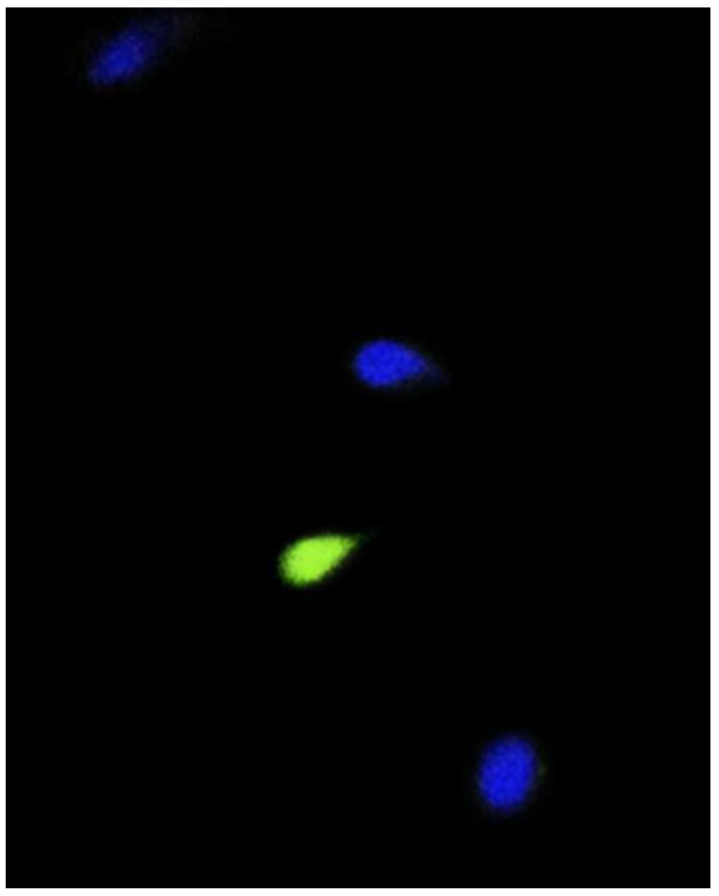
Intracellular ROS (green cell) in human sperm cell analyzed by fluorescent microscopy using DCFH_2_-DA probe.

The intracellular sperm levels of ROS were measured by DCF assay with a 2,7-dichlorodihydrofluorescein diacetate (DCFH_2_-DA) probe, as described in Santonastaso and collaborators [9]. Briefly, 13 μm DCFH_2_-DA was added to 150 µL sperm suspension in MEM. After 30 min incubation at 37 °C in the dark, the cell suspension was washed with bicarbonate buffer and counterstained with 4′,6-Diamidine-2′-phenylindole dihydrochloride (DAPI) solution for 5 min. Then, the sperm cells were transferred to a glass slide and observed by using a fluorescent microscope (Nikon Eclipse E-600) equipped with BP 330–380 nm and LP 420 nm filters. Intracellular ROS were visually scored comparing the control and samples and were measured as the percentage of sperm cells exhibiting a response (green cells) on the total sperm cells (Figure 3). The DCF assay was performed in triplicate.

### 2.8. Statistical Analysis

All sperm parameters and the experimental data were expressed as the mean ± standard deviation (SD). Differences in the percentage of DNA damage, GTS%, DFI% and intracellular ROS% among the experimental groups were compared using the unpaired Student’s *t*-test by GraphPad Prism 6. The effect was considered significant if *p*-value ≤ 0.01 with respect to the negative control.

## 3. Results

### 3.1. Sperm Motility Is Reduced after 90 min TiO_2_NPs and CdCl_2_ Co-Exposure

CdCl_2_ exposure and TiO_2_NPs-CdCl_2_ co-exposure did not cause a statistically significant reduction in vitality. CdCl_2_ treatment induced a statistically significant reduction of motility (progressive and non-progressive) after 30 min, while TiO_2_NPs-CdCl_2_ co-exposure reduced sperm motility after 90 min (*p*-value ≤ 0.01) (Table 3).

### 3.2. TiO_2_NPs and CdCl_2_ Co-Exposure Causes Time-Dependent Loss of Sperm DNA Integrity 

**Figure 4:**
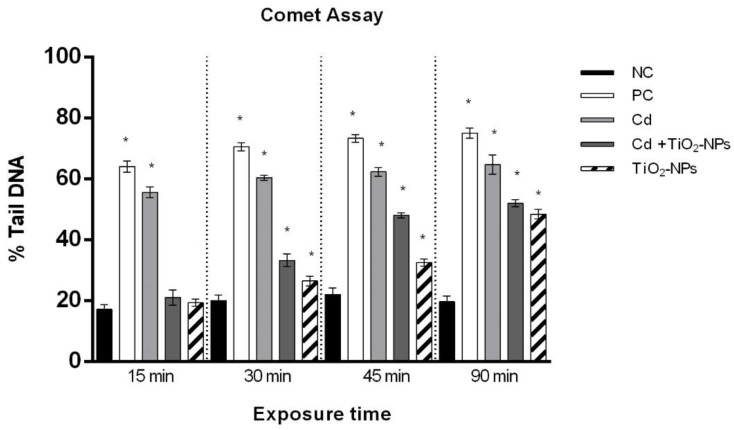
Percentage of DNA in the comet tail (ordinate) in human sperm after different exposure times (abscissa) to CdCl_2_ and CdCl_2_ + TiO_2_NPs co-exposure. The black bars are negative controls (NC); the white bars are positive controls (PC) (benzene 0.4 μL/mL); the light grey bars are 10 µM CdCl_2_-treated sperm (Cd); the dark grey bars are 10 µM CdCl_2_ + 1 μg/L TiO_2_NPs-treated sperm (Cd + TiO_2_NPs); the striped bars are 1 μg/L TiO_2_NPs-treated sperm. * *p* ≤ 0.01.

The results of the Comet assay showed that TiO_2_NPs-CdCl_2_ co-exposure induced a time-dependent loss of human sperm DNA integrity with statistically significant values (*p*-value ≤ 0.01) after 30 min. Furthermore, CdCl_2_ exposure already reduced the sperm DNA integrity after 15 min (Figure 4).

### 3.3. TiO_2_NPs and CdCl_2_ Combined Exposure Induces Sperm DNA Fragmentation

**Figure 5:**
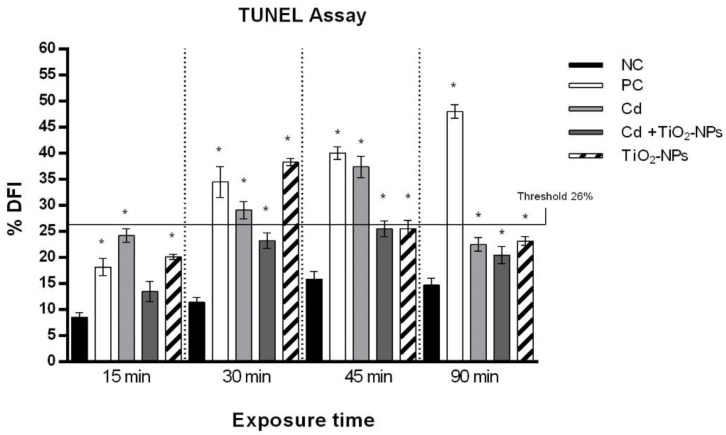
Percentage of DNA fragmentation index (ordinate) in human sperm after different exposure times (abscissa) to CdCl_2_ and CdCl_2_ + TiO_2_NPs co-exposure. The black bars are negative controls (NC); the white bars are positive controls (PC) (benzene 0.4 μL/mL); the light grey bars are 10 µM CdCl_2_-treated sperm (Cd); the dark grey bars are 10 µM CdCl_2_ + 1 μg/L TiO_2_NPs-treated sperm (Cd + TiO_2_NPs); the striped bars are 1 μg/L TiO_2_NPs-treated sperm. * *p* ≤ 0.01.

The data from the TUNEL test displayed a statistically significant (*p*-value ≤ 0.01) increase of sperm DNA fragmentation starting from 30 min of TiO_2_NPs and CdCl_2_ co-exposure, whereas the CdCl_2_ single exposure induced sperm DNA fragmentation starting as early as after 15 min. The DNA Fragmentation Index (DFI) corresponding to the cut-off value (26%) associated to male infertility [53] was not exceeded after the TiO_2_NPs and CdCl_2_ combined exposure (Figure 5).

### 3.4. TiO_2_NPs in Combination with CdCl_2_ Produce Intracellular Oxidative Stress in Sperm Cells

**Figure 6:**
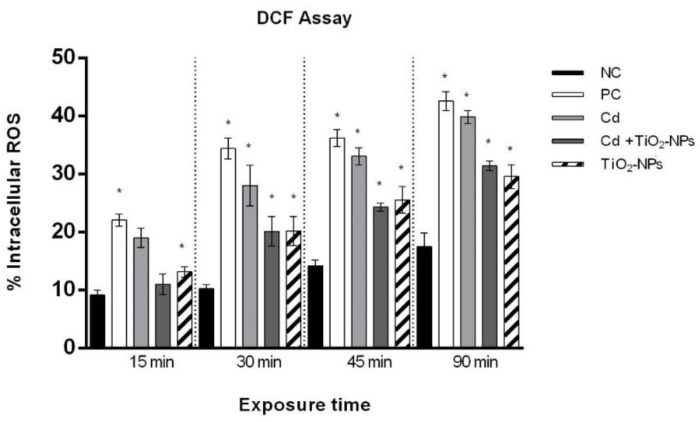
Percentage of intracellular ROS (ordinate) in human sperm after different exposure times (abscissa) to CdCl_2_ and CdCl_2_ + TiO_2_NPs co-exposure. The black bars are negative controls (NC); the white bars are positive controls (PC) (benzene 0.4 μL/mL); the light grey bars are 10 µM CdCl_2_-treated sperm (Cd); the dark grey bars are 10 µM CdCl_2_ + 1 μg/L TiO_2_NPs-treated sperm (Cd + TiO_2_NPs); the striped bars are 1 μg/L TiO_2_NPs-treated sperm. * *p* ≤ 0.01.

A statistically significant increase (*p*-value ≤ 0.01) of intracellular ROS was observed in sperm cells exposed to CdCl_2_ alone starting from 15 min and in combination with TiO_2_NPs starting from 30 min with respect to the control. The increasing intracellular oxidative stress was time-dependent after co-exposure to CdCl_2_ and TiO_2_NPs (Figure 6).

### 3.5. TiO_2_NPs and CdCl_2_ Co-Exposure Generates Sperm DNA Polymorphic Profiles Alterations

The RAPD-PCR analysis showed a variation of polymorphic profiles of sperm DNA exposed to CdCl_2_ alone and in combination with TiO_2_NPs compared to non-treated sperm DNA. CdCl_2_ treatment induced the appearance of one band and the disappearance of two bands with respect to the control after 15 min, while after 30 of exposure mins three news bands appeared and one band disappeared. After 45 exposure mins we evidenced the appearance of three news bands and the disappearance of two bands with respect to the control. The exposure to CdCl_2_ for 90 mins induced the prevalence of bands’ disappearance when compared to the control. The electrophoretic patterns relative to the TiO_2_NPs-CdCl_2_ co-exposure showed the prevalence of bands’ appearance when compared to non-treated sperm cells (Table 4).

### 3.6. TiO_2_NPs in Combination with CdCl_2_ Reduce Sperm Genome Stability

**Figure 7:**
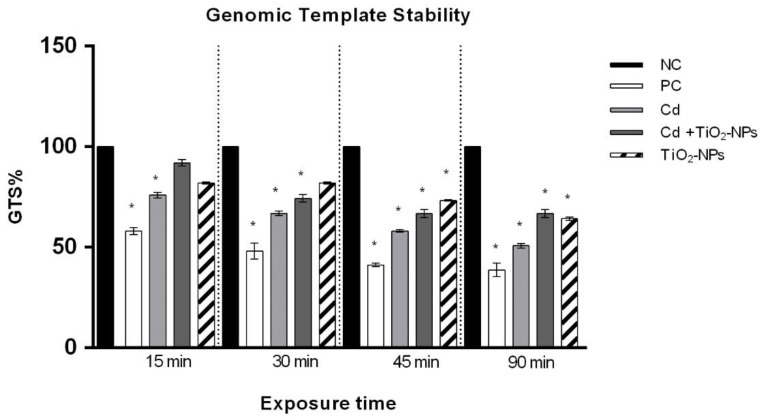
Changes in the percentage of Genome Template Stability in human sperm DNA (ordinate) after different exposure times (abscissa) to CdCl_2_ and CdCl_2_+TiO_2_NPs co-exposure, as evidenced by the RAPD-PCR technique. The black bars are the negative controls (NC); the white bars are the positive controls (PC) (benzene 0.4 μL/mL); the light grey bars are 10 µM CdCl_2_-treated sperm (Cd); the dark grey bars are 10 µM CdCl_2_ + 1 μg/L TiO_2_NPs-treated sperm (Cd + TiO_2_NPs); the striped bars are 1 μg/L TiO_2_NPs-treated sperm. *p ≤ 0.01.

The human sperm genome stability (GTS%) significantly decreased in the CdCl_2_-exposed sperms in a time-dependent manner. The genome stability of co-exposed sperms was statistically reduced after 30 min (Figure 7).

## 4. Discussion

The chemical–physical characteristics of TiO_2_NPs make them capable of absorbing and transporting numerous compounds through biological barriers, including Cd, with a mechanism known as the “Trojan Horse” effect. The transport can take place either by simple diffusion from the caveola systems or by endocytosis with transport mediated by ABC family proteins [14].

The effects of NPs’ and heavy metals’ co-exposure on the living organism are still unclear. Data on heavy metal and TiO_2_NPs genotoxicity and cytotoxicity are controversial, especially because these interactions are species-specific, often tissue-specific and related to physical–chemical features of the co-exposure medium [22,54,55]. The aim of our work was to examine in vitro the genotoxic responses induced by Cd alone and in association with TiO_2_NPs in human sperm cells at different times (15, 30, 45 and 90 min).

Scarce amounts of data are available regarding TiO_2_NPs’ effects on reproduction/fertility. In adult male Wistar rats, TiO_2_NPs’ daily oral exposure (50 mg/kg body weight (BW)/day) caused significant time-dependent adverse effects such as decreased testis and prostate weight, disrupted hormone profile (i.e., significant decreased serum testosterone level and increased serum estradiol, Luteinizing hormone (LH) and Follicle stimulating hormone (FSH) levels), impaired spermatogenesis, lipid peroxidation and inflammation in testicular tissues. Moreover, effects on semen parameters were also reported: normal sperm counts decreased from 88% (control) to 68% after 21 days of exposure [56]. Song and collaborators [57] examined the testes and sperm quality in male mice after an oral 5–10 nm TiO_2_NP exposure to of 0, 10, 50 and 100 mg/kg BW for 28 days. TiO_2_NPs exposure caused sperm malformations, a sperm cell micronucleus rate and levels of markers indicating cell damage in the testes, a further reduction in the germ cell number and spherospermia, interstitial glands, malalignment and vacuolization in spermatogenic cells at the two highest dose levels (50 and 100 mg/kg BW). The superoxide dismutase (SOD) activity significantly decreased at the highest dose level (100 mg/kg BW) and the malondialdehyde significantly increased at the two highest dose levels (50 and 100 mg/kg BW), both of which are markers indicating cell damage in testis, although the weights of the testicles and epididymis were not affected. Conversely, no effect on male reproductive parameters (weight of reproductive organs, daily sperm production and plasma testosterone levels) was reported for adult male mice after weekly TiO_2_NP intratracheal instillation for seven weeks [58].

Our previous study showed that TiO_2_NPs cause a loss of sperm DNA integrity and a statistically significant increase in DNA fragmentation and DNA strand breaks, inducing apoptosis [9]. In males, apoptosis plays a physiological role by maintaining an appropriate germ cell to Sertoli cell ratio, removing defective germ cells and controlling sperm production [59]; however, elevated apoptosis levels can damage the spermatozoa. Our results showed that TiO_2_NPs were genotoxic on human sperm cells in vitro, significantly affecting the reproductive potential.

Furthermore, exposure to Cd induced apoptosis in rats and frog testes, rat Leydig cells, trophoblast cells of rat placenta and granulosa cells of chicken ovarian follicles [60,61,62,63,64]. This heavy metal, commonly present in the environment in the form of CdCl_2_, is able to alter sperms’ motility and their capacity to reach and penetrate into the oocyte by altering the sperm enzyme acetylcholine transferase and the oxygen uptake [35].

Sperms’ acute exposure to Cd may impair the sperm fertilization potential in vitro; in fact, exposure to CdCl_2_ results in a decreased progressive and hyperactivated sperm motility, as well as increased caspase activation, which suggests the triggering of an apoptotic pathway [36]. In vitro Cd-exposed murine spermatozoa exhibit an altered sperm fertilization potential, producing a lower number of pronuclei than controls during in vitro fertilization [37]. A severe reduction of sperm motility and kinematic parameters was also shown in a rat model exposed to Cd in vivo [38].

Our results showed that exposure to CdCl_2_ caused a reduction of human sperm motility in a time-dependent manner and a decrease of the genome template stability, which was associated with an increased level of DNA strand breaks, apoptosis and oxidative stress. These results confirmed the genotoxic potential of Cd though the induction of an oxidative microenvironment [65]. Our results showed that the presence of TiO_2_NPs can reduce the cytotoxicity and genotoxicity associated with single Cd exposure as sperm showed a higher motility, and a lower induction of DNA strand breaks as well as of the apoptotic pathway after co-exposure highlighted by the Comet assay. We also estimated human sperm DNA fragmentation by TUNEL test. The sperm DNA fragmentation index (DFI) is considered a valuable early marker of the presence and harmful effects of pollution [66]. A pathological parameter (26%) was correlated with the inability to fertilize the egg cell, and was thus associated with poliabortivity [67]. Cd and TiO_2_NPs co-exposure induced a lower sperm DNA fragmentation than that observed by single Cd exposure for all exposure times, and the pathological value of DFI was never exceeded; otherwise, Cd caused an increase beyond the pathological value after 30 and 45 exposure mins. Sperm DNA fragmentation decreased with an increasing exposure time, suggesting that sperms could undergo increasing damage of the genetic material in the first exposure times (15, 30 and 45 min), but there was a decrease of the sperm DNA fragmentation at 90 min, thus probably implying that sperms activated the cellular repair mechanisms, as evidenced by the Comet assay, at an exposure time longer than 45 min.

Alterations in RAPDs’ profiles have allowed for the detection of genomic instability as different molecular events (genomic rearrangements, point mutations, deletions and insertions) of sperm treated with respect to the sperm negative control. In fact, the appearance of a new band is related to point mutations and/or rearrangements, while the disappearance of the band is attributable to DNA adducts and breaks in the double helix [68]. The RAPD-PCR analysis was able to detect DNA changes not necessarily related to apoptotic processes. We observed that CdCl_2_ treatment induced both the appearance of new bands and disappearance of bands in comparison with the control, while CdCl_2_-TiO_2_NPs co-exposure resulted in the prevalence of new bands’ appearance with respect to the non-treated sperm cells as well as those induced by treatment with 1 μg/L TiO_2_NPs, as previously reported by Santonastaso and collaborators [9]. Thanks to RAPDs’ profile alterations, we evaluated the sperm genomic template stability percentage (%GTS). The decrease of %GTS depends on the time exposure to single Cd and Cd-TiO_2_NPs. The decrease of %GTS is the first molecular response to a toxicant and has been demonstrated as being directly related to the extent of DNA damage and/or to the efficiency of DNA repair and replication [7,69]. Our study showed a statistically significant decrease in genomic stability in sperm exposed to Cd already after 15 min, which reached deep values after 90 exposure minutes, while co-exposure significantly impacted genomic stability after only 30 min.

The changes in RAPDs’ profiles highlighted in sperm cells exposed to Cd alone and in association with TiO_2_NPs could be related to oxidative DNA damage through ROS direct damage on genetic material [70,71]. To clarify this assumption, we assessed intracellular ROS production in both experimental conditions. Co-exposure resulted in lower intracellular ROS levels when compared to single Cd exposure, indicating that ROS detrimental effects could be the main mechanism for Cd and TiO_2_NPs’ genotoxicity.

The results showed that sperm DNA damage probably due to ROS action induced by Cd-TiO_2_NPs co-exposure is lower than in the case of Cd single exposure, and we may speculate that the Cd genotoxic potential was inhibited and/or masked by TiO_2_NPs; thus, no “Trojan Horse” effect was demonstrated in human spermatozoa in vitro. Unfortunately, evidence on transport, absorption, agglomeration and reactivity in the synergy between nanoparticles and Cd is still scarce and contradictory. Contrary to our results, other studies reported that TiO_2_NPs potentiated Cd-induced pro-oxidants generation (ROS and lipid peroxidation), antioxidants depletion (glutathione level and glutathione reductase, SOD and catalase enzymes) and apoptosis (by altering the gene expression of p53, bax and bcl-2), along with the alteration of the mitochondrial membrane potential in HepG2 and MCF-7 cells [72]. Xia and collaborators [73] reported that TiO_2_NPs may promote Cd-induced cellular oxidative stress in human embryo kidney 293T (HEK293T) cells, as indicated by the changes in the SOD activity and ROS concentration. Cd and TiO_2_NPs exert additive or synergistic effects on HEK293T cells’ oxidative damage, and these effects vary with different proportions and concentrations of CdCl_2_ and TiO_2_NPs in the mixture. TiO_2_NPs assume different behaviors depending on features of the exposure medium and duration of exposure. In salt water, TiO_2_NPs begin to form aggregates such as sedimentations after about 6 h; it was also highlighted that the presence of CdCl_2_ increases the aggregation between the nanoparticles and their sedimentation [12]. However, when the size of TiO_2_NPs is around 23.8 nm, they do not form visible and appreciable aggregates, independently of the duration of exposure in fresh water [7].

Nanoparticles show a strong tendency to form agglomerates in solution due to their high surface area [74]. The agglomerate (or cluster) is defined as a compound formed by the secondary aggregates (which are joined by weak chemical bonds), which can be broken through manipulation. The state of dispersion of a particulate system describes the relative number of primary particles (aggregated) present in a suspension medium. The general view is that the degree and type of agglomerates formed may influence the toxicity of NPs. The aggregate can also be defined as a compound of the secondary primary particles (which are joined by means of strong chemical bonds), which behaves as a single unit [75]. Our results by TEM evidenced that TiO_2_NP agglomerates in the range of 400 ± 52 nm do not penetrate into the human sperm cells. They exercise a pro-oxidant effect on the polyunsaturated fatty acids of the sperm membrane, resulting in intracellular ROS generation and the induction of different DNA damage degrees, depending on the endpoints investigated in the sperm exposure medium (MEM) [9,15,46,76]. In this study, we demonstrated that sperm genotoxicity induced by TiO_2_NPs and CdCl_2_ co-exposure significantly decreased when compared to that induced by Cd. Therefore, we can speculate that TiO_2_NPs and Cd could form complexes, whose size does not allow them to penetrate into the sperm cells. Moreover, the degree and the type of aggregates could influence TiO_2_NPs’ and Cd’s sperm genotoxicity in MEM. In industrial wastewaters, the presence of TiO_2_NPs and Cd^2+^ results in the formation of a ternary surface complex with arsenic that inhibits Cd release into the aqueous phase and that, hence, facilitates the immobilization of the heavy metal [77]. As shown in the Quantitative Structure-Activity Relationship (QSAR) computational method [77], the interaction between TiO_2_NPs and Cd can result in the formation of a “sandwich structure”, where Cd, placed at the center, is completely masked by the TiO_2_NPs, which are located at the outer surfaces of the structures. It can be concluded that TiO_2_NPs–CdCl_2_ co-exposure could lead to the formation of aggregates with a reduced genotoxic activity due to their sandwich organization [46].

Based on our knowledge, this is the first study reporting the genotoxic effects of the combined exposure to TiO_2_NPs and CdCl_2_ of human sperm cells in vitro. The association of the two tested contaminants leads to a reduction in the single molecules’ genotoxic effects, probably due to the formation of aggregates, as predicted by the in silico analysis. Although these results could be interpreted as positive in relation to health risks, we must underline that our data are limited to an in vitro system; hence, we cannot draw any conclusion on a systemic impact of the co-exposure. Therefore, considering the widespread existence of these contaminants in the environment, further studies are necessary in order to clarify the pathways that are responsible for TiO_2_NPs and CdCl_2_ genotoxicity and to better clarify their potential interaction.

## Figures and Tables

**Table 1:** Dispersion of tested 1 μg/L TiO_2_NPs-enriched culture media at 0, 15, 30, 45 and 90 min calculated on the TiO_2_NPs calibration curve, obtained by plotting the absorbance at the maximum wavelength (325 nm) vs. the different sonicated standard solutions’ dose levels.

Time (min)	nTiO_2_NPs [1 μg/L]
0	1.01 ± 0.01
15	0.91 ± 0.05
30	0.87 ± 0.02
45	0.69 ± 0.01
90	0.27 ± 0.03

**Table 2:** Parameters of semen fluid selected for the study. Sperm parameters were expressed as mean ± SD.

Sperm Parameters	Mean± SD
Semen volume (mL)	3.5 ± 0.5
Ph	6.9 ± 0.3
Sperm concentration (*10^6^ sperm/mL)	60.4 ± 15.6
Vitality (%)	70.8 ± 5.8
Motility (%)	
Progressive	69.0 ± 15.7
Non-progressive	20.0 ± 5.5
Immobile	11 ± 9
Normal morphology (%)	15 ± 6.5

**Table 3:** Sperm vitality and motility after CdCl_2_ exposure and TiO_2_NPs co-exposure. The values were expressed as mean ± SD. * *p* ≤ 0.01.

Substances Concentration	Exposure Minutes	Vitality (%)	Motility (P + NP) (%)
CdCl_2_ 10 µM µg/L	15 30 45 90	71 ± 4.5 65 ± 2.5 65 ± 5.6 61 ± 4.5	81 ± 5.5 69 ± 8.0 * 57 ± 5.8 * 55 ± 7.5 *
CdCl_2_ 10 µM + TiO_2_NPs 1 µg/L	15 30 45 90	72 ± 3.2 70 ± 2.7 69 ± 4.1 65 ± 4.5	78 ± 6.9 74 ± 8.5 70 ± 8.7 58 ± 5.5 *

**Table 4:** Molecular sizes (bp) of appeared and disappeared bands after amplification with primer D11 in human sperm DNA exposed to CdCl_2_ and CdCl_2_ with TiO_2_NPs co-exposure. * Control bands are at: 190, 270, 450, 500, 510, 560, 650, 850, 910, 950, 1000 and 2000 bp.

Substances Concentration	Exposure Minutes	Gained Bands	Lost Bands *
CdCl_2_ 10 µM µg/L	15 30 45 90	210 300, 350, 900 210, 700, 900 600	560, 850 560 270,850 270, 450, 500, 560, 850
CdCl_2_ 10 µM + TiO_2_NPs 1 µg/L	15 30 45 90	700 210, 350, 900 210, 530, 900 300, 400, 900	- - 850 850
